# Supplementary material for: Chicken Hypothalamic and Ovarian DNA Methylome Alteration in Response to Forced Molting
Source: Animals (Basel). 2023 Mar 10;13(6):1012. doi: 10.3390/ani13061012 (PMC10044502; doi:10.3390/ani13061012)
Supplement: Supplementary file 1 [file animals-13-01012-s001.zip › Supplementary_Materials.pdf]

Supplementary Material

Table S1 Statistical analysis for genome-wide DNA methylation sequencing data

Samples	Raw Reads No. (10^6)	Clean Reads No. (10^6)	N (%) (10^{-4})	Q20 (%)	Q30 (%)	Mapping rate (%)
1-1-h	207.57	194.95	3.62	95.26	89.42	87.42
1-1-o	198.94	185.00	2.81	94.93	89.12	89.66
1-2-h	252.75	240.04	3.60	95.43	89.22	87.17
1-2-o	257.64	229.35	2.78	93.32	87.22	87.13
1-3-h	198.41	187.64	3.63	95.35	89.28	88.90
1-3-o	219.17	200.25	2.86	94.24	88.07	89.99
2-1-h	215.91	205.48	3.60	95.44	89.23	89.42
2-1-o	207.98	190.66	2.79	93.73	87.33	89.40
2-2-h	255.97	242.89	3.60	95.27	89.03	88.44
2-2-o	201.76	190.63	2.81	95.53	89.55	87.90
2-3-h	285.94	270.01	3.64	95.51	89.58	84.55
2-3-o	205.54	192.99	2.80	94.98	88.90	87.26
3-1-h	266.11	189.92	4.20	95.36	89.29	90.81
3-1-o	211.14	178.33	4.24	94.84	88.60	90.65
3-2-h	225.12	204.88	4.19	95.57	89.66	88.86
3-2-o	236.00	192.22	4.20	95.20	89.38	90.92
3-3-h	256.75	193.91	4.20	95.65	89.84	88.39
3-3-o	259.27	199.33	4.22	95.49	89.58	91.07
4-1-h	199.21	170.42	6.62	94.82	88.21	86.60
4-1-o	186.41	204.49	5.06	94.48	87.56	90.07
4-2-h	214.32	198.53	4.98	94.65	87.93	90.89
4-2-o	202.12	192.68	6.34	94.35	87.65	92.16
4-3-h	212.69	208.73	7.62	95.19	88.59	88.26
4-3-o	209.87	176.54	6.85	94.84	88.45	91.29
5-1-h	222.63	212.72	13.12	95.38	89.53	90.64
5-1-o	214.89	199.83	7.61	94.88	88.35	89.87

5-2-h	213.99	207.46	6.38	95.49	89.22	90.62
5-2-o	223.05	203.17	6.13	94.46	87.75	88.72
5-3-h	244.23	218.40	7.48	95.02	88.36	87.30
5-3-o	246.91	185.97	6.19	94.75	88.28	88.98

Note: Sample: The sample names;
Reads No.: The total reads;
N(%): the percentage of fuzzy bases;
Q20(%): The percentage of bases with a base recognition accuracy of more than 99%;
Q30(%): The percentage of bases with a base recognition accuracy of more than 99.9%;
Mapping rate (%): the rate of reads mapped to chicken reference genomes;
i-j-o (i=1,2,3,4,5; j=1,2,3) stand for three biological replicates from five periods in ovarian samples;
i-j-h (i=1,2,3,4,5; j=1,2,3) stand for three biological replicates from five periods in hypothalamic samples;

Table S2 The proportion of methylated C sites in CG, CHG and CHH contexts of 30 samples from forced molting

Samples	mC（%)	mCG（%)	mCHG（%)	mCHH（%)
1-1-h	4.39	88.36	1.70	9.94
1-1-o	3.72	93.23	1.07	5.70
1-2-h	4.53	90.92	1.36	7.72
1-2-o	3.44	93.86	1.03	5.10
1-3-h	4.30	89.60	1.58	8.82
1-3-o	3.93	93.38	1.07	5.54
2-1-h	4.59	78.23	3.36	18.41
2-1-o	4.03	93.47	1.08	5.45
2-2-h	4.67	90.92	1.37	7.71
2-2-o	4.17	93.84	1.02	5.14
2-3-h	4.65	90.69	1.40	7.91
2-3-o	4.24	93.87	1.02	5.10
3-1-h	4.32	92.94	1.11	5.96
3-1-o	3.04	94.45	0.94	4.61
3-2-h	3.27	91.43	1.29	7.28
3-2-o	4.22	94.15	1.00	4.85
3-3-h	4.38	92.16	1.20	6.64
3-3-o	3.87	94.49	0.94	4.57
4-1-h	3.56	91.78	1.26	6.96
4-1-o	4.38	94.14	0.99	4.87
4-2-h	4.16	91.50	1.30	7.20
4-2-o	4.36	94.12	0.99	4.89
4-3-h	1.42	91.98	1.24	6.78
4-3-o	1.60	94.07	1.00	4.93
5-1-h	4.81	76.45	3.68	19.86

5-1-o	4.06	93.85	1.00	5.15
5-2-h	4.42	91.97	1.22	6.81
5-2-o	4.35	94.04	1.01	4.95
5-3-h	4.57	91.36	1.29	7.35
5-3-o	4.48	93.88	1.01	5.11

Note: mC (%) represents the percentage of methylated C sites out of the total number of C sites in the whole genome. The mCG (%), mCHG (%) and mCHH (%) represent the proportion of methylated C sites in all methylated C sites from different sequence environments, where H stands for A, C and T.

Table S9 The DMGs associated with cellular aging in three different periods

1-vs-2	hypothalamus	hyper_DMGs	SYNJ1 [1], UBE3A [2,3], ARHGEF7 [4]
	hypothalamus	hypo_DMGs	ATAD2B [5], RARS2 [6], WNT9A [7]
	ovary	hyper_DMGs	DIP2C [8], DOCK7 [9], HSF1 [10]
	ovary	hypo_DMGs	BRSK1 [11], KCNK2 [12], UBE2D3 [13]
2-vs-3	hypothalamus	hyper_DMGs	ABCA1 [14-16], CCPG1 [17],COL4A2 [18]
	hypothalamus	hypo_DMGs	GADD45A [19], HOXA3 [20], MEIS1 [21]
	ovary	hyper_DMGs	DNAJC1 [22], FBXL7 [23,24], KIF5C [25], PANK3 [26]
	ovary	hypo_DMGs	DCLK1 [27-29]
3-vs-5	hypothalamus	hyper_DMGs	ATOH8 [30], NUMB [31], POT1[32]
	hypothalamus	hypo_DMGs	SEPT5 [33], ZEB2 [34-36], YAP1 [37,38], RXRA [39]
	ovary	hyper_DMGs	CEP128 [40], EXOC4 [41], ZDHHC13 [42-44]
	ovary	hypo_DMGs	BIN3 [45], CPXM1 [46], RARB [47,48], RPS15 [49]

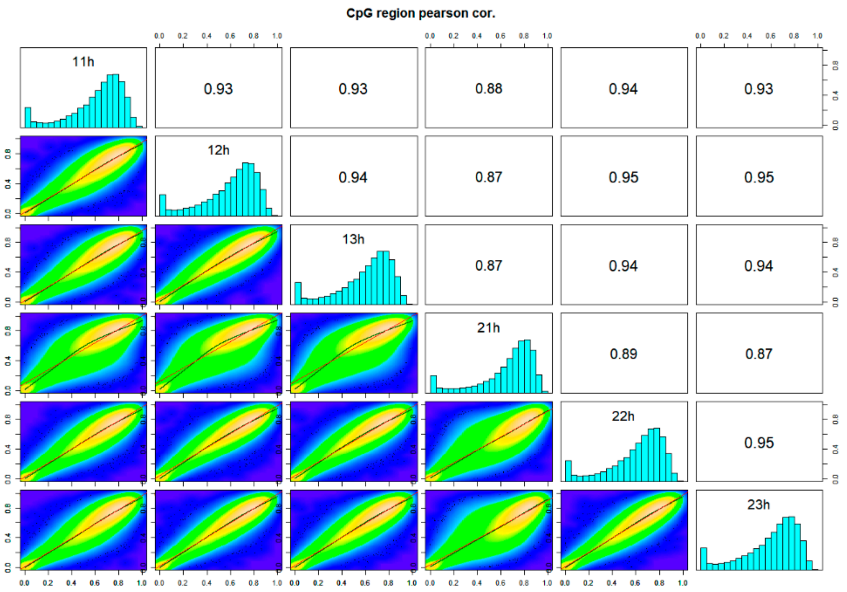

Figure S1. The CpG region correlation of samples for 1-vs-2 group in hypothalamus

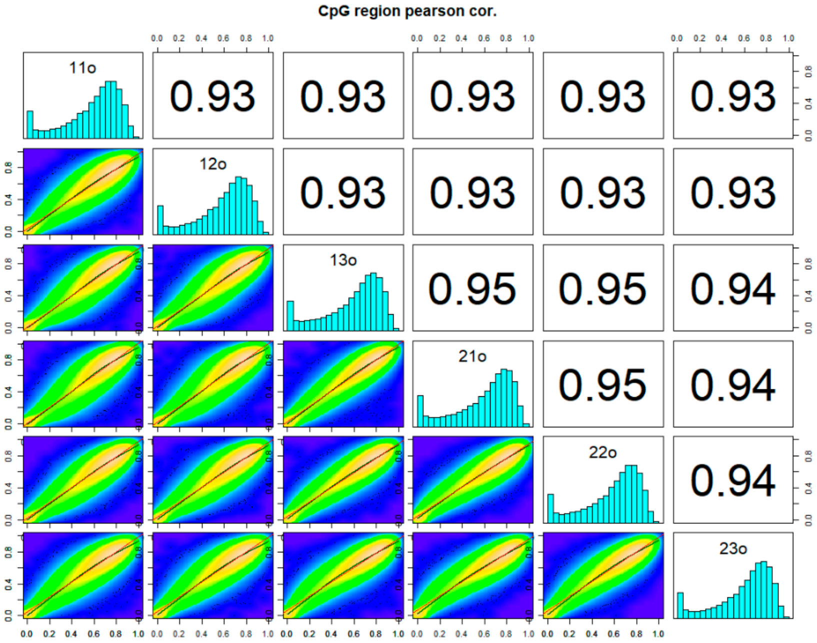

Figure S2. The CpG region correlation of samples for 1-vs-2 group in ovary

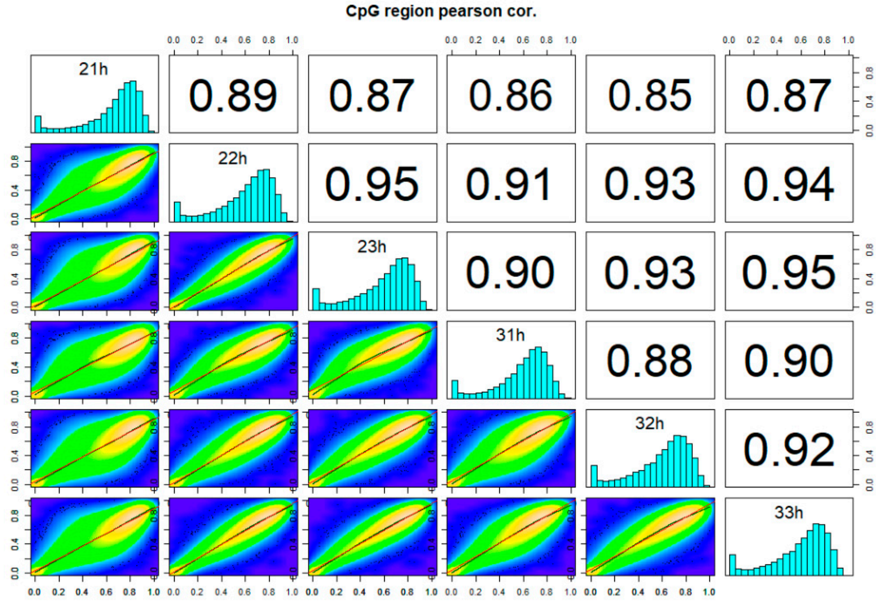

Figure S3. The CpG region correlation of samples for 2-vs-3 group in hypothalamus

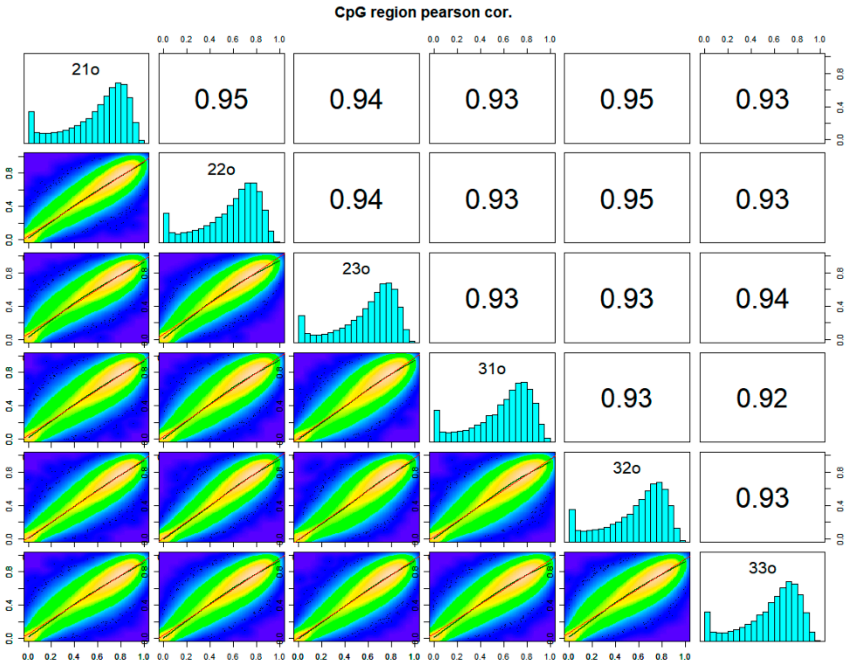

Figure S4. The CpG region correlation of samples for 2-vs-3 group in ovary

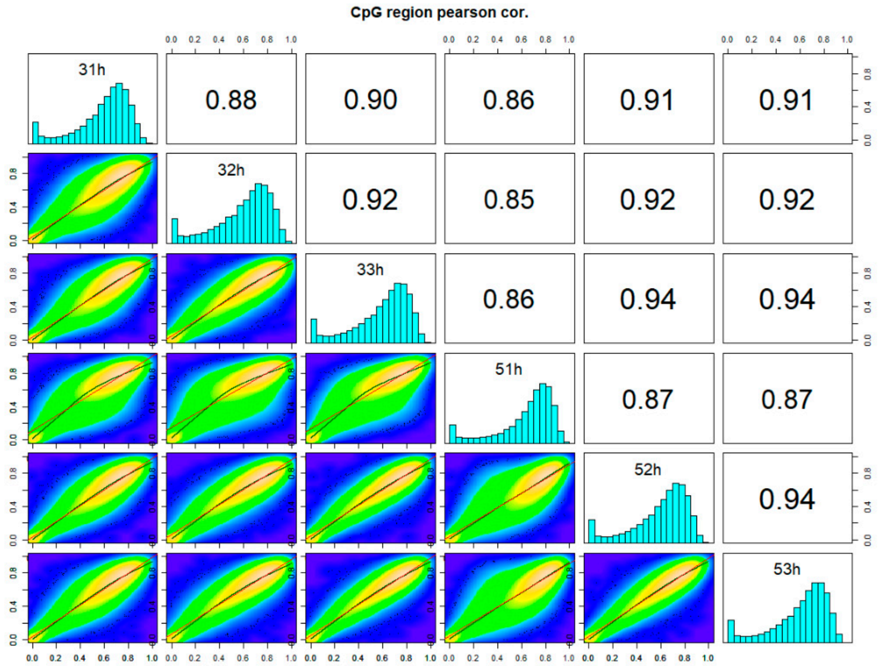

Figure S5. The CpG region correlation of samples for 3-vs-5 group in hypothalamus

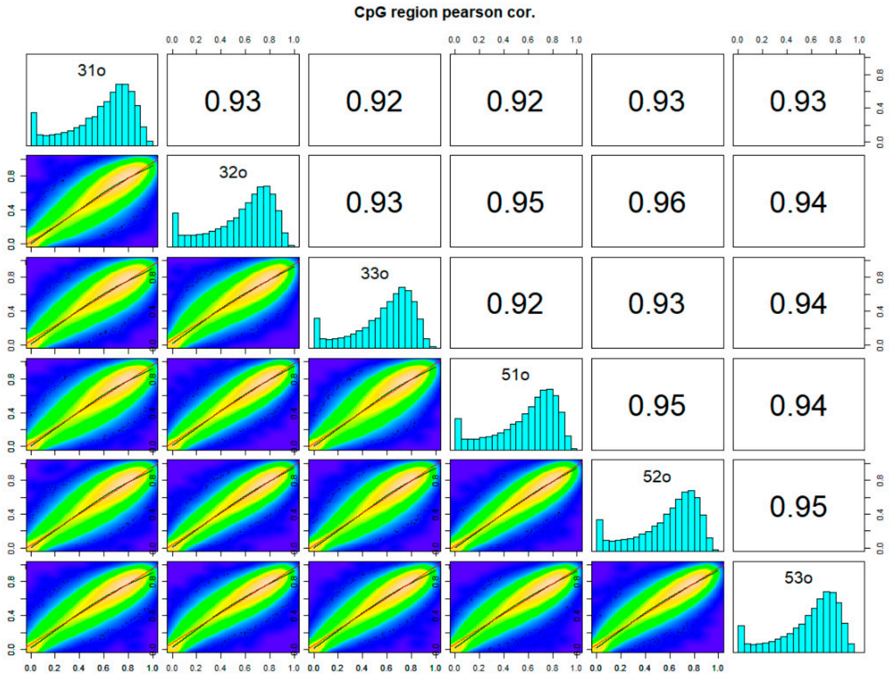

Figure S6. The CpG region correlation of samples for 3-vs-5 group in ovary

References

1. Samanta, D. & Arya, K. Electroclinical Findings of SYNJ1 Epileptic Encephalopathy. *J*

Pediatr Neurosci. 2020, 15, 29-33.
2. Vatsa, N. & Jana, N. R. UBE3A and Its Link With Autism. *Front Mol Neurosci*. 2018, 11, 448.
3. Elgersma, Y. & Sonzogni, M. UBE3A reinstatement as a disease-modifying therapy for Angelman syndrome. *Dev Med Child Neurol.* 2021, 63, 802-807.
4. López Tobón, A., Suresh, M., Jin, J., Vitriolo, A., Pietralla, T., Tedford, K., Bossenz, M., Mahnken, K., Kiefer, F., Testa, G., Fischer, K. D. & Püschel, A. W. The guanine nucleotide exchange factor Arhgef7/βPix promotes axon formation upstream of TC10. *Sci Rep*. 2018, 8, 8811.
5. Leachman, N. T., Brellier, F., Ferralli, J., Chiquet-Ehrismann, R. & Tucker, R. P. ATAD2B is a phylogenetically conserved nuclear protein expressed during neuronal differentiation and tumorigenesis. *Dev Growth Differ*. 2010, 52, 747-55.
6. Ngoh, A., Bras, J., Guerreiro, R., Meyer, E., McTague, A., Dawson, E., Mankad, K., Gunny, R., Clayton, P., Mills, P. B., Thornton, R., Lai, M., Forsyth, R. & Kurian, M. A. RARS2 mutations in a sibship with infantile spasms. *Epilepsia*. 2016, 57, e97-e102.
7. Luo, C., Zhou, S., Zhou, Z., Liu, Y., Yang, L., Liu, J., Zhang, Y., Li, H., Liu, Y., Hou, F. F. & Zhou, L. Wnt9a Promotes Renal Fibrosis by Accelerating Cellular Senescence in Tubular Epithelial Cells. *J Am Soc Nephrol*. 2018, 29, 1238-1256.
8. Larsson, C., Ali, M. A., Pandzic, T., Lindroth, A. M., He, L. & Sjöblom, T. Loss of DIP2C in RKO cells stimulates changes in DNA methylation and epithelial-mesenchymal transition. *BMC cancer*. 2017, 17, 487.
9. Le, P. T., Bishop, K. A., Maridas, D. E., Motyl, K. J., Brooks, D. J., Nagano, K., Baron, R., Bouxsein, M. L. & Rosen, C. J. Spontaneous mutation of Dock7 results in lower trabecular bone mass and impaired periosteal expansion in aged female Misty mice. *Bone*. 2017, 105, 103-114.
10. Kruta, M., Sunshine, M. J., Chua, B. A., Fu, Y., Chawla, A., Dillingham, C. H., Hidalgo San Jose, L., De Jong, B., Zhou, F. J. & Signer, R. A. J. Hsf1 promotes hematopoietic stem cell fitness and proteostasis in response to ex vivo culture stress and aging. *Cell stem cell*. 2021, 28, 1950-1965.e6.
11. Qin, Y., Sun, M., You, L., Wei, D., Sun, J., Liang, X., Zhang, B., Jiang, H., Xu, J. & Chen, Z. J. ESR1, HK3 and BRSK1 gene variants are associated with both age at natural menopause and premature ovarian failure. *Orphanet J Rare Dis*. 2012, 7, 5.
12. Innamaa, A., Jackson, L., Asher, V., van Schalkwyk, G., Warren, A., Keightley, A., Hay, D., Bali, A., Sowter, H. & Khan, R. Expression and effects of modulation of the K2P potassium channels TREK-1 (KCNK2) and TREK-2 (KCNK10) in the normal human ovary and epithelial ovarian cancer. *Clin Transl Oncol*. 2013 15, 910-8.
13. Gibson, J., Russ, T. C., Clarke, T. K., Howard, D. M., Hillary, R. F., Evans, K. L., Walker, R. M., Bermingham, M. L., Morris, S. W., Campbell, A., Hayward, C., Murray, A. D., Porteous, D. J., Horvath, S., Lu, A. T., McIntosh, A. M., Whalley, H. C. & Marioni, R. E. A meta-analysis of genome-wide association studies of epigenetic age acceleration. *PLoS Genet*. 2019, 15, e1008104.
14. Guay, S. P., Légaré, C., Houde, A. A., Mathieu, P., Bossé, Y. & Bouchard, L. Acetylsalicylic acid, aging and coronary artery disease are associated with ABCA1 DNA methylation in men. *Clin Epigenetics*. 2014, 6, 14.
15. Mockute, R., Vilkeviciute, A., Balciuniene, V. J., Zemaitiene, R. & Liutkeviciene, R. ABCA1 rs1883025 and CYP4F2 rs2108622 Gene Polymorphism Association with Age-Related Macular Degeneration and Anti-VEGF Treatment. *Medicina*. 2021, 57(9):974.
16. Tian, M., Zhang, X., Wang, L. & Li, Y. Curcumin induces ABCA1 expression and apolipoprotein A-I-mediated cholesterol transmembrane in the chronic cerebral hypoperfusion aging rats. *Am J Chin Med*. 2013, 41, 1027-42.
17. Zohar, K., Lezmi, E., Eliyahu, T. & Linial, M. Ladostigil Attenuates Induced Oxidative Stress in Human Neuroblast-like SH-SY5Y Cells. *Biomedicines*. 2021, 9(9):1251.

18. Kanazawa, Y., Nagano, M., Koinuma, S., Sugiyo, S. & Shigeyoshi, Y. Effects of aging on basement membrane-related gene expression of the skeletal muscle in rat. *Biomed Res*. 2021, 42(3):115-119.
19. Diao, D., Wang, H., Li, T., Shi, Z., Jin, X., Sperka, T., Zhu, X., Zhang, M., Yang, F., Cong, Y., Shen, L., Zhan, Q., Yan, J., Song, Z. & Ju, Z. Telomeric epigenetic response mediated by Gadd45a regulates stem cell aging and lifespan. *EMBO Rep*. 2018, 19(10): e45494.
20. Turner, D. C., Gorski, P. P., Maasar, M. F., Seaborne, R. A., Baumert, P., Brown, A. D., Kitchen, M. O., Erskine, R. M., Dos-Remedios, I., Voisin, S., Eynon, N., Sultanov, R. I., Borisov, O. V., Larin, A. K., Semenova, E. A., Popov, D. V., Generozov, E. V., Stewart, C. E., Drust, B., Owens, D. J., Ahmetov, II & Sharples, A. P. DNA methylation across the genome in aged human skeletal muscle tissue and muscle-derived cells: the role of HOX genes and physical activity. *Sci Rep*. 2020, 10(1):15360.
21. Chang-Panesso, M., Kadyrov, F. F., Machado, F. G., Kumar, A. & Humphreys, B. D. Meis1 is specifically upregulated in kidney myofibroblasts during aging and injury but is not required for kidney homeostasis or fibrotic response. *Am J Physiol-Renal*. 2018, 315, F275-f290.
22. Lindsey, J. C., Lusher, M. E., Strathdee, G., Brown, R., Gilbertson, R. J., Bailey, S., Ellison, D. W. & Clifford, S. C. Epigenetic inactivation of MCJ (DNAJD1) in malignant paediatric brain tumours. *Int J Cancer*. 2006, 118(2):346-52.
23. Moro, L. & Pagano, M. Epigenetic suppression of FBXL7 promotes metastasis. *Mol Cell Oncol*. 2020, 7(6):1833698..
24. Chiu, H. W., Chang, J. S., Lin, H. Y., Lee, H. H., Kuei, C. H., Lin, C. H., Huang, H. M. & Lin, Y. F. FBXL7 Upregulation Predicts a Poor Prognosis and Associates with a Possible Mechanism for Paclitaxel Resistance in Ovarian Cancer. *J Clin Med*. 2018, 7(10):330.
25. Dathe, V., Pröls, F. & Brand-Saberi, B. Expression of kinesin kif5c during chick development. *Anat Embryol*. 2004, 207, 475-80.
26. Li, J., Li, C., Li, Q., Li, G., Li, W., Li, H., Kang, X. & Tian, Y. Novel Regulatory Factors in the Hypothalamic-Pituitary-Ovarian Axis of Hens at Four Developmental Stages. *Front Genet*. 2020. 11, 591672.
27. Cheng, L., Yang, Z., Guo, W., Wu, C., Liang, S., Tong, A., Cao, Z., Thorne, R. F., Yang, S. Y., Yu, Y. & Chen, Q. DCLK1 autoinhibition and activation in tumorigenesis. *Innovation*. 2020, 3, 100191.
28. Nevi, L., Di Matteo, S., Carpino, G., Zizzari, I. G., Samira, S., Ambrosino, V., Costantini, D., Overi, D., Giancotti, A., Monti, M., Bosco, D., De Peppo, V., Oddi, A., De Rose, A. M., Melandro, F., Bragazzi, M. C., Faccioli, J., Massironi, S., Grazi, G. L., Panici, P. B., Berloco, P. B., Giuliante, F., Cardinale, V., Invernizzi, P., Caretti, G., Gaudio, E. & Alvaro, D. DCLK1, a Putative Stem Cell Marker in Human Cholangiocarcinoma. *Hepatology*. 2021, 73, 144-159.
29. Liu, W., Wang, S., Sun, Q., Yang, Z., Liu, M. & Tang, H. DCLK1 promotes epithelial-mesenchymal transition via the PI3K/Akt/NF-κB pathway in colorectal cancer. *Int J Cancer*. 2018, 142, 2068-2079.
30. Keder, A., Tardieu, C., Malong, L., Filia, A., Kashkenbayeva, A., Newton, F., Georgiades, M., Gale, J. E., Lovett, M., Jarman, A. P. & Albert, J. T. Homeostatic maintenance and age-related functional decline in the Drosophila ear. *Sci Rep*. 2020, 10, 7431.
31. De Gasperi, R., Mo, C., Azulai, D., Wang, Z., Harlow, L. M., Du, Y., Graham, Z., Pan, J., Liu, X. H., Guo, L., Zhang, B., Ko, F., Raczkowski, A. M., Bauman, W. A., Goulbourne, C. N., Zhao, W., Brotto, M. & Cardozo, C. P. Numb is required for optimal contraction of skeletal muscle. *J Cachexia Sarcopenia Muscle*. 2022, 13, 454-466.
32. Li, T., Luo, Z., Lin, S., Li, C., Dai, S., Wang, H., Huang, J., Ma, W., Songyang, Z. & Huang, Y. MiR-185 targets POT1 to induce telomere dysfunction and cellular senescence. *Aging*. 2020, 12, 14791-14807.
33. Khairat, J. E., Balasubramaniam, V., Othman, I., Omar, A. R. & Hassan, S. S. Interaction of

Recombinant Gallus gallus SEPT5 and Brain Proteins of H5N1-Avian Influenza Virus-Infected Chickens. *Proteomes*. 2017, 5(3):23.
34. Epifanova, E., Babaev, A., Newman, A. G. & Tarabykin, V. Role of Zeb2/Sip1 in neuronal development. *Brain Res.* 2019, 1705, 24-31.
35. Huang, X., Ferris, S. T., Kim, S., Choudhary, M. N. K., Belk, J. A., Fan, C., Qi, Y., Sudan, R., Xia, Y., Desai, P., Chen, J., Ly, N., Shi, Q., Bagadia, P., Liu, T., Guilliams, M., Egawa, T., Colonna, M., Diamond, M. S., Murphy, T. L., Satpathy, A. T., Wang, T. & Murphy, K. M. Differential usage of transcriptional repressor Zeb2 enhancers distinguishes adult and embryonic hematopoiesis. *Immunity*. 2021, 54, 1417-1432.e7.
36. Hegarty, S. V., Sullivan, A. M. & O'Keeffe, G. W. Zeb2: A multifunctional regulator of nervous system development. *Prog Neurobiol*. 2015, 132, 81-95.
37. Chen, X., Li, Y., Luo, J. & Hou, N. Molecular Mechanism of Hippo-YAP1/TAZ Pathway in Heart Development, Disease, and Regeneration. *Front Physiol*. 2020, 11, 389.
38. Molina, L., Nejak-Bowen, K. & Monga, S. P. Role of YAP1 Signaling in Biliary Development, Repair, and Disease. *Semin Liver Dis*. 2022, 42, 17-33.
39. Ma, X., Warnier, M., Raynard, C., Ferrand, M., Kirsh, O., Defossez, P. A., Martin, N. & Bernard, D. The nuclear receptor RXRA controls cellular senescence by regulating calcium signaling. *Aging cell*. 2018, 17, e12831.
40. Li, R., Yang, Y. E., Yin, Y. H., Zhang, M. Y., Li, H. & Qu, Y. Q. Methylation and transcriptome analysis reveal lung adenocarcinoma-specific diagnostic biomarkers. *J Transl Med*. 2019, 17, 324.
41. He, Y., Zhou, X., Zheng, R., Jiang, Y., Yao, Z., Wang, X., Zhang, Z., Zhang, H., Li, J. & Yuan, X. The Association of an SNP in the EXOC4 Gene and Reproductive Traits Suggests Its Use as a Breeding Marker in Pigs. *Animals*. 2021, 11(2):521.
42. Shen, L. F., Chen, Y. J., Liu, K. M., Haddad, A. N. S., Song, I. W., Roan, H. Y., Chen, L. Y., Yen, J. J. Y., Chen, Y. J., Wu, J. Y. & Chen, Y. T. Role of S-Palmitoylation by ZDHHC13 in Mitochondrial function and Metabolism in Liver. *Sci Rep.* 2017, 7, 2182.
43. Liu, Y., Xu, Q., Kang, X., Wang, K., Wang, J., Feng, D., Bai, Y. & Fang, M. Dynamic changes of genomic methylation profiles at different growth stages in Chinese Tan sheep. *J Anim Sci Biotechnol.* 2021, 12, 118.
44. Cao, Y., Jin, H. G., Ma, H. H. & Zhao, Z. H. Comparative analysis on genome-wide DNA methylation in longissimus dorsi muscle between Small Tailed Han and Dorper×Small Tailed Han crossbred sheep. *Asian-Australas J Anim Sci*. 2017, 30, 1529-1539.
45. Ramalingam, A., Duhadaway, J. B., Sutanto-Ward, E., Wang, Y., Dinchuk, J., Huang, M., Donover, P. S., Boulden, J., McNally, L. M., Soler, A. P., Muller, A. J., Duncan, M. K. & Prendergast, G. C. Bin3 deletion causes cataracts and increased susceptibility to lymphoma during aging. *Cancer Res*. 2008, 68, 1683-90.
46. Zheng, M., Long, J., Chelariu-Raicu, A., Mullikin, H., Vilsmaier, T., Vattai, A., Heidegger, H. H., Batz, F., Keckstein, S., Jeschke, U., Trillsch, F., Mahner, S. & Kaltofen, T. Identification of a Novel Tumor Microenvironment Prognostic Signature for Advanced-Stage Serous Ovarian Cancer. *Cancers*. 2021, 13(13):3343.
47. Losi-Guembarovski, R., Kuasne, H., Guembarovski, A. L., Rainho, C. A. & Cólus, I. M. DNA methylation patterns of the CDH1, RARB, and SFN genes in choroid plexus tumors. *Cancer Genet Cytogenet*. 2007, 179, 140-5.
48. Udhane, S. S. & Flück, C. E. Regulation of human (adrenal) androgen biosynthesis-New insights from novel throughput technology studies. *Biochem Pharmacol*. 2016, 102, 20-33.
49. Wu, S., Xu, S., Li, R., Li, K., Zhong, X., Li, Y., Zhou, Z., Liu, Y., Feng, R., Zheng, J., Songyang, Z. & Liu, F. mTORC1-Rps15 Axis Contributes to the Mechanisms Underlying Global Translation Reduction During Senescence of Mouse Embryonic Fibroblasts. *Front Cell Dev Biol*. 2019, 7, 337.
